# Sirt1 Protects against Oxidative Stress-Induced Apoptosis in Fibroblasts from Psoriatic Patients: A New Insight into the Pathogenetic Mechanisms of Psoriasis

**DOI:** 10.3390/ijms19061572

**Published:** 2018-05-25

**Authors:** Matteo Becatti, Victoria Barygina, Amanda Mannucci, Giacomo Emmi, Domenico Prisco, Torello Lotti, Claudia Fiorillo, Niccolò Taddei

**Affiliations:** 1 Department of Experimental and Clinical Biomedical Sciences “Mario Serio”, University of Florence, 50134 Florence, Italy; matteo.becatti@unifi.it (M.B.); v.barygina@gmail.com (V.B.); amanda.mannucci@unifi.it (A.M.); niccolo.taddei@unifi.it (N.T.); 2 Department of Experimental and Clinical Medicine, University of Florence, 50134 Florence, Italy; giacomo.emmi@unifi.it (G.E.); domenico.prisco@unifi.it (D.P.); 3 Department of Dermatology, University of Rome “G. Marconi”, 00146 Rome, Italy; professor@torellolotti.it

**Keywords:** SIRT1, MAPK, oxidative stress, psoriasis

## Abstract

Psoriasis, a multisystem chronic disease characterized by abnormal keratinocyte proliferation, has an unclear pathogenesis where systemic inflammation and oxidative stress play mutual roles. Dermal fibroblasts, which are known to provide a crucial microenvironment for epidermal keratinocyte function, represented the selected experimental model in our study which aimed to clarify the potential role of SIRT1 in the pathogenetic mechanisms of the disease. We firstly detected the presence of oxidative stress (lipid peroxidation and total antioxidant capacity), significantly reduced SIRT1 expression level and activity, mitochondrial damage and apoptosis (caspase-3, -8 and -9 activities) in psoriatic fibroblasts. Upon SIRT1 activation, redox balance was re-established, mitochondrial function was restored and apoptosis was no longer evident. Furthermore, we examined p38, ERK and JNK activation, which was strongly altered in psoriatic fibroblasts, in response to SIRT1 activation and we measured caspase-3 activity in the presence of specific MAPK inhibitors demonstrating the key role of the SIRT1 pathway against apoptotic cell death via MAPK modulation. Our results clearly demonstrate the involvement of SIRT1 in the protective mechanisms related to fibroblast injury in psoriasis. SIRT1 activation exerts an active role in restoring both mitochondrial function and redox balance via modulation of MAPK signaling. Hence, SIRT1 can be proposed as a specific tool for the treatment of psoriasis.

## 1. Introduction

Psoriasis, a chronic, inflammatory multisystemic disease affecting 2–3% of the world population, is characterized by abnormal keratinocyte proliferation resulting in the formation of raised, itchy and well-demarcated erythematous lesions on the skin. The pathogenesis of psoriasis is not well understood, but recent data suggest that both innate and adaptive immunity have a key role in the disease [1]. Aberrant activation and metabolism of epidermal keratinocytes leading to strongly enhanced keratinocyte proliferation and anomalous terminal differentiation are the hallmark of psoriatic skin. The critical role of dermal fibroblasts in providing a crucial microenvironment for epidermal keratinocyte function [2] and in regulating epidermal morphogenesis was already suggested [3]. Moreover, recent reports demonstrated that, in vivo, improvement of aged dermal fibroblast function significantly increases epidermal keratinocyte proliferation with consequent enlargement of epidermal thickness [4]. However, the molecular features and the factors responsible for the complex interactions between dermal stromal cells and epidermal keratinocytes in both physiologic and pathologic conditions are still to be elucidated. On these bases, we focused on dermal fibroblasts which exert specific functions in the microenvironment of epidermal keratinocytes and, together with infiltrating PMNs, induce a marked redox imbalance in psoriatic derma by extensively producing reactive oxygen species (ROS), such as superoxide and H_2_O_2_, and thus displaying a significant role in the anomalous keratinocyte growth which characterizes psoriasis [5].

The key role of ROS in the pathogenesis of psoriasis has been already reported [6,7]. Indeed, several studies have revealed increased levels of oxidative stress markers and decreased activity of the main antioxidant enzymes in the plasma of psoriatic patients [8,9,10,11,12]. In addition, it has been demonstrated that mitogen-activated protein kinases (MAPK) pathways, such as extracellular signal-regulated kinase (ERK), c-Jun N-terminal kinase (JNK) and p38 MAPK, are involved in the pathogenesis of psoriasis [7,13,14,15,16,17]. However, the molecular regulatory mechanisms of these cellular signal transduction pathways and their possible interplay are not fully elucidated. A variety of stress stimuli, including oxidative stress, induces functional changes of these kinases similar to those found to be associated with apoptosis [18]. Similarly, NAD^+^-dependent protein deacetylase SIRT1 has been shown to be strongly upregulated by oxidative stress [19]. SIRT1, the most extensively studied member of the sirtuin family, plays a key role in metabolism, stress responses, and many other cellular processes [20]. Additionally, our previous data demonstrated that SIRT1 upregulation affects the MAPK pathway and inhibits pro-apoptotic molecules, reducing oxidative stress and apoptosis in several cellular models [21,22].

Here, for the first time, we investigated in primary fibroblasts from lesional psoriatic skin, the possible involvement of SIRT1 in MAPK pathways.

## 2. Results

### 2.1. SIRT1 Expression and Activity in Fibroblasts from Healthy and Psoriatic Subjects

**Figure 1:**
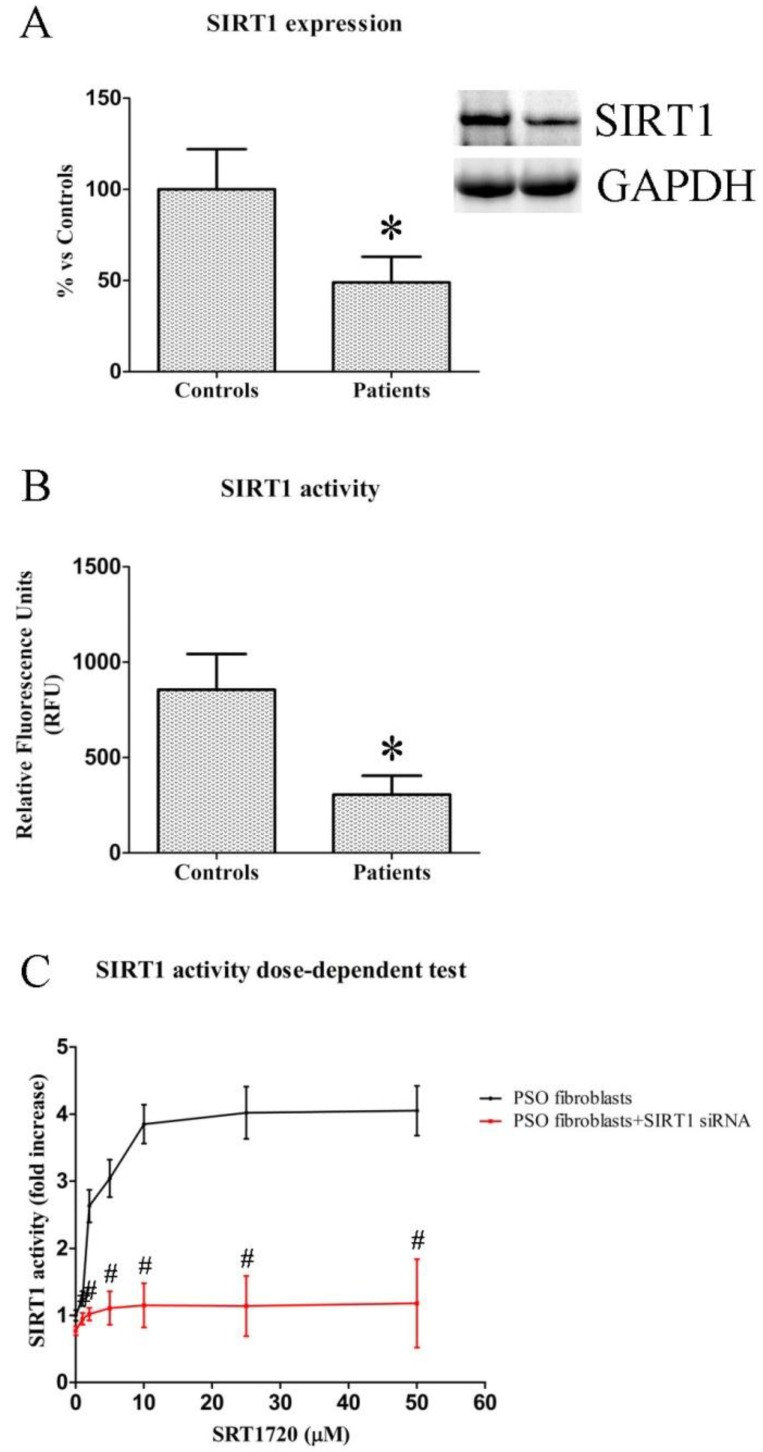
(**A**) Representative Western blot analysis of SIRT1 expression in fibroblasts from controls and psoriatic patients. Histogram represents data from controls (*n* = 4 biopsies) and patients (*n* = 4 biopsies); (**B**) SIRT1 activity in fibroblasts from controls (*n* = 4 biopsies) and psoriatic patients (*n* = 4 biopsies); (**C**) SIRT1 activity in fibroblasts from lesional psoriatic skin (*n* = 4 biopsies) after 24 h of incubation with different concentrations of SRT1720. Each experiment was performed in triplicate. * Significant difference (*p* ≤ 0.05) vs. fibroblasts from healthy patients. # Significant difference (*p* ≤ 0.01) vs. PSO fibroblasts.

We evaluated SIRT1 expression and activity in fibroblasts from healthy and psoriatic patients. As previously demonstrated [23], SIRT1 expression (Figure 1A) was markedly reduced in fibroblasts from psoriatic patients compared to healthy subjects (−51% vs. control fibroblasts, *p* < 0.01). Similarly, SIRT1 activity (Figure 1B) in psoriatic fibroblasts exhibited a significant decrease in comparison with healthy cells (306 ± 99 vs. 855 ± 188, *p* < 0.01).

### 2.2. Dose-Dependent Effects of SIRT1720 on SIRT1 Activity in Psoriatic Fibroblasts

A dose-dependent test was performed in psoriatic fibroblasts treated with SRT1720 concentrations from 1 to 50 µM (Figure 1C) to evaluate the effect of SRT1720 on SIRT1 activity. Treatment (24 h) with 10 µM SRT1720 induced a dramatic increase in SIRT1 activity (3.85 ± 0.29 fold increase). Hence, 10 µM SRT1720 was used for the programmed experiments. Interestingly, the addition of SIRT1 siRNA to SRT1720-treated cells induced a complete abolishment of the observed increase (*p* < 0.01) (Figure 1C).

### 2.3. SIRT1 Activation Decreases Oxidative Stress in Fibroblasts from Psoriatic Patients

**Figure 2:**
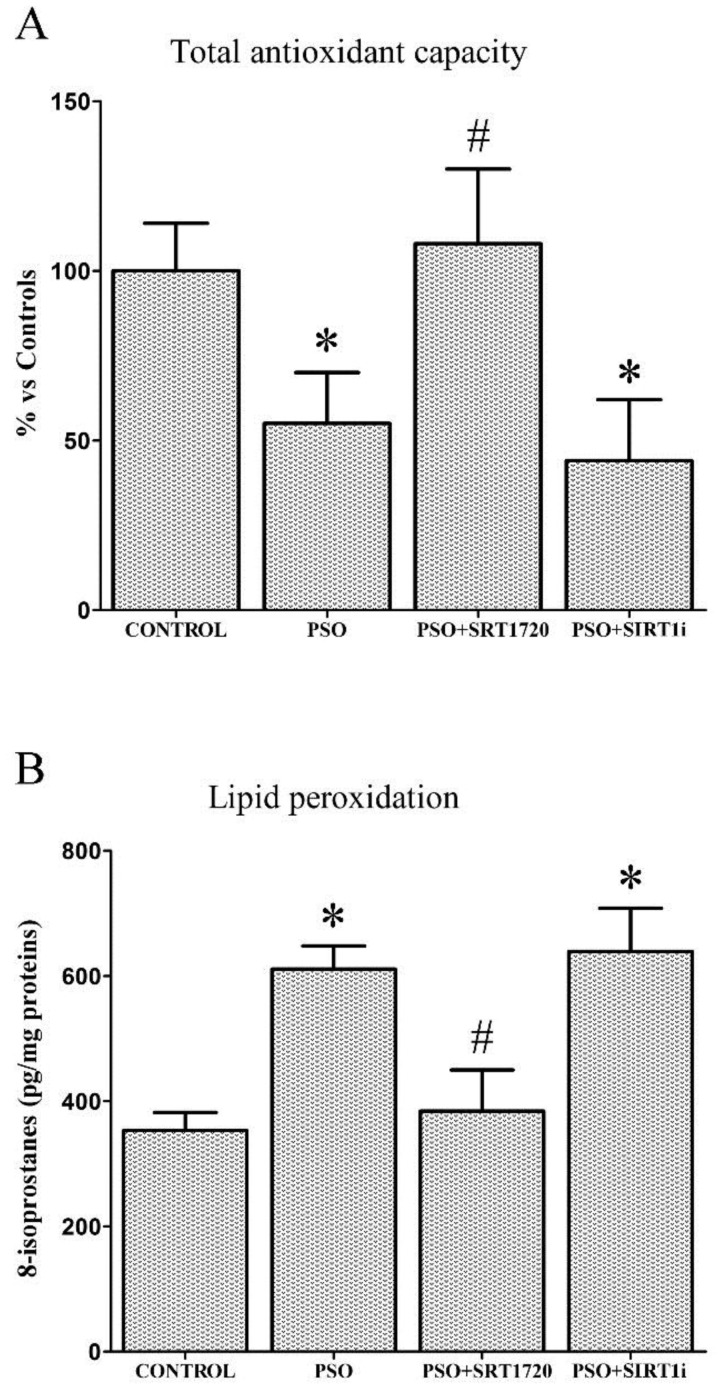
(**A**) Evaluation of total antioxidant capacity (TAC) and (**B**) 8-isoprostanes in fibroblasts from controls (*n* = 4 biopsies) and psoriatic patients (*n* = 4 biopsies) in the presence of SRT1720 or the SIRT1 inhibitor (SIRT1i). Each experiment was performed in triplicate. * Significant difference (*p* ≤ 0.05) vs. fibroblasts from healthy patients. # Significant difference (*p* ≤ 0.05) vs. fibroblasts from psoriatic patients.

Figure 2A shows a significant total antioxidant capacity (TAC) decrease in psoriatic fibroblasts with respect to controls (−45%, *p* < 0.01).

**Figure 3:**
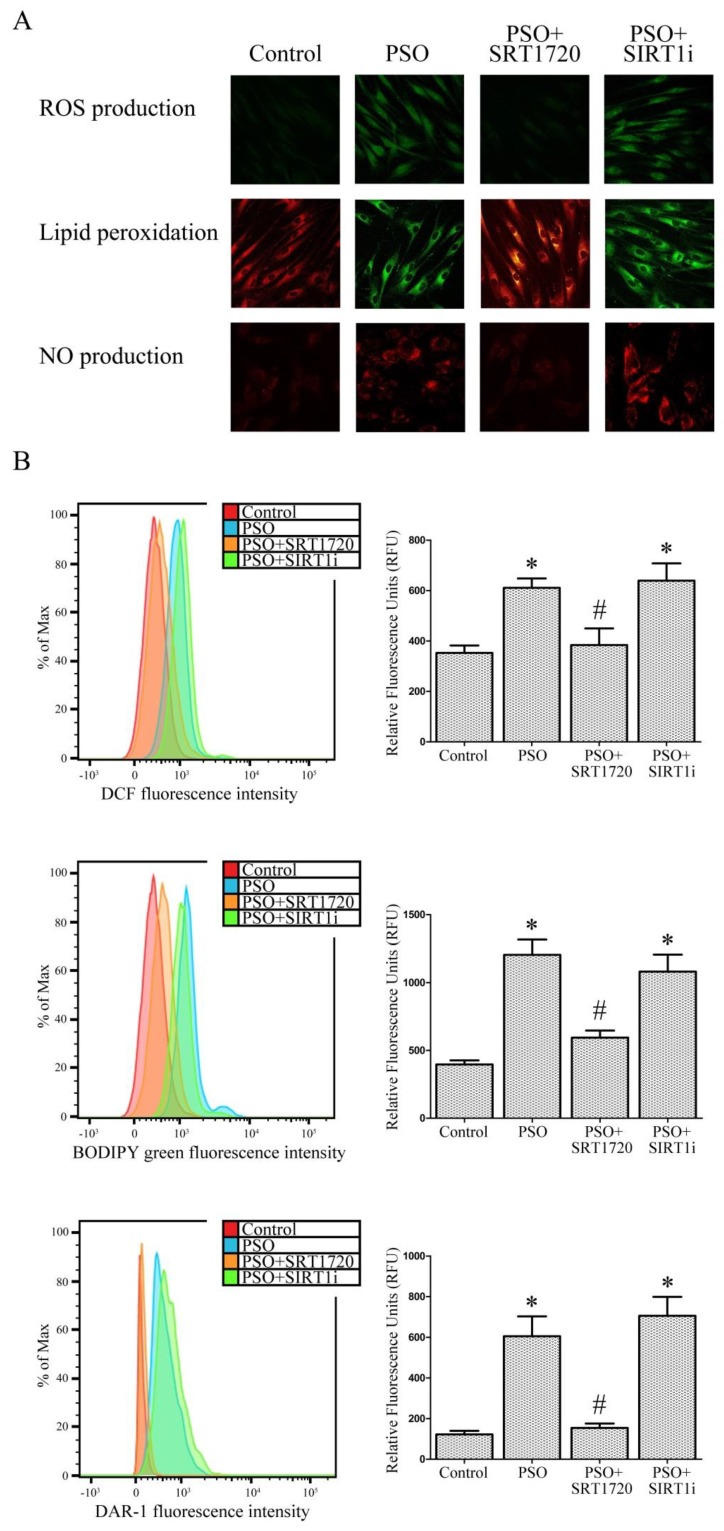
(**A**) Confocal microscope analysis (63× magnification) and (**B**) FACS analysis of ROS production, lipoperoxidation and NO production in fibroblasts from controls (*n* = 4 biopsies) and psoriatic patients (*n* = 4 biopsies) in the presence of SRT1720 or the SIRT1 inhibitor (SIRT1i). Each experiment was performed in triplicate. * Significant difference (*p* ≤ 0.05) vs. fibroblasts from healthy patients. # Significant difference (*p* ≤ 0.05) vs. fibroblasts from psoriatic patients.

SIRT1 activation effectively restored intracellular TAC levels (+53% vs. untreated PSO cells, *p* < 0.01); interestingly, this effect was abrogated by the SIRT1 inhibitor, demonstrating the key role of SIRT1 in improving antioxidant defense systems. Increased levels of 8-isoprostanes (lipid peroxidation markers) were also found in psoriatic fibroblasts with respect to control fibroblasts (+73%, *p* < 0.01). SRT1720-treated psoriatic fibroblasts showed significantly lower 8-isoprostanes levels (−47% vs. untreated PSO cells, *p* < 0.01), confirming the pivotal role of SIRT1 pathways in cell redox balance (Figure 2B). Similar results were found when lipid peroxidation was measured using BODIPY by flow cytometry and confocal microscopy (Figure 3). Similarly, the fluorescent probe H_2_DCFDA was used for determining intracellular ROS production (Figure 3). SRT1720-treated fibroblasts displayed less marked ROS production, thus indicating a strong protective effect exerted by SIRT1 pathways against ROS. Analogous results were found when we evaluated NO production (Figure 3).

### 2.4. SIRT1 Activation Protects Psoriatic Fibroblasts from Mitochondrial Damage

**Figure 4:**
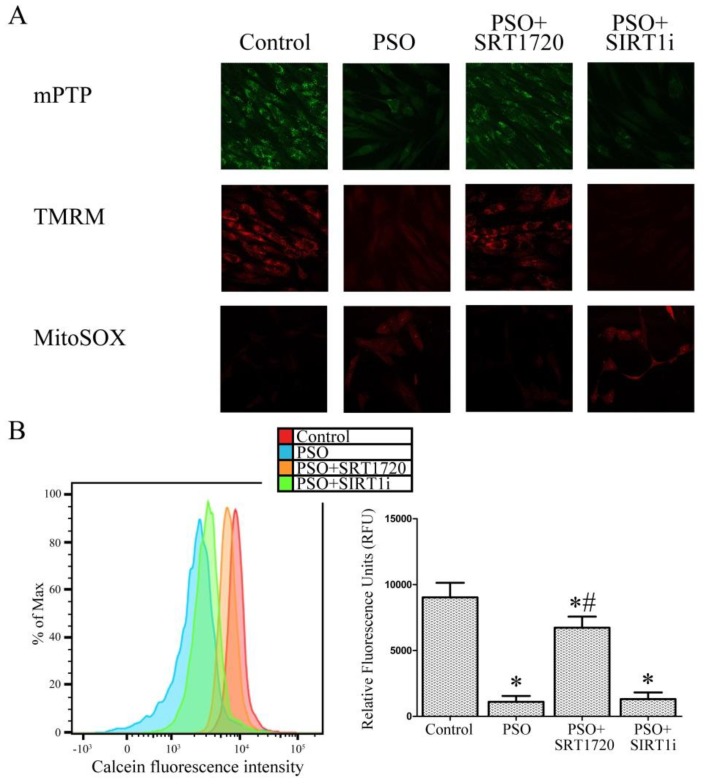

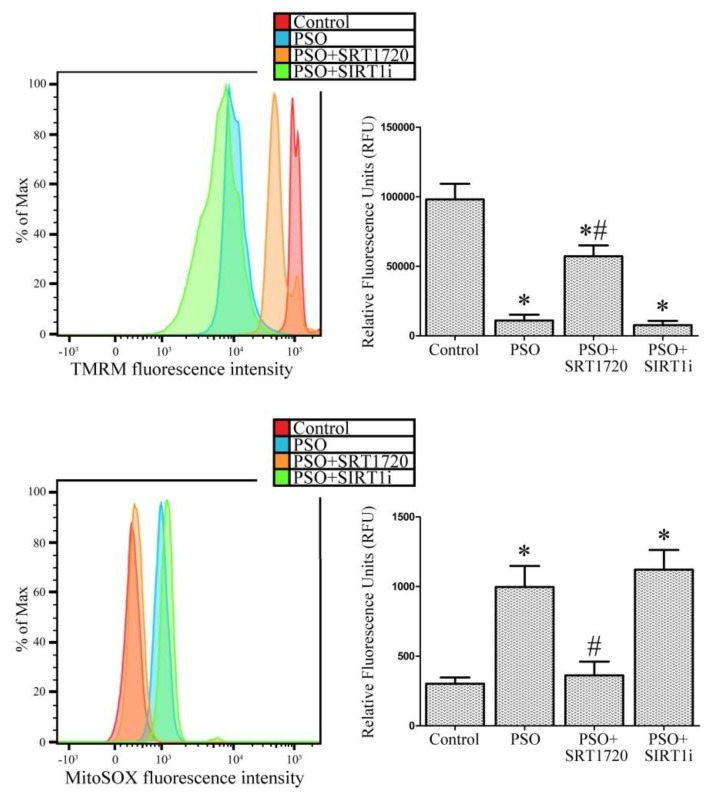
(**A**) Confocal microscope analysis (63× magnification) and (**B**) FACS analysis of mitochondrial permeability transition pore opening (mPTP), mitochondrial depolarisation (TMRM) and mitochondrial superoxide production (MitoSOX) in fibroblasts from controls (*n* = 4 biopsies) and psoriatic patients (*n* = 4 biopsies) in the presence of SRT1720 or the SIRT1 inhibitor (SIRT1i). Each experiment was performed in triplicate. * Significant difference (*p* ≤ 0.05) vs. fibroblasts from healthy patients. # Significant difference (*p* ≤ 0.05) vs. fibroblasts from psoriatic patients.

In order to ascertain whether SIRT1 activation can protect against mitochondrial damage, we analyzed the mitochondrial permeability transition pore opening, mitochondrial membrane polarization and mithocondrial superoxide production. Confocal microscope analysis (Figure 4A) indicated marked alterations in mitochondrial permeability transition pore (mPTP) opening and mitochondrial membrane depolarization (TMRM probe) in fibroblasts from psoriatic patients. These changes were not evident in control cells. SIRT1 activation by SRT1720 efficiently restored mitochondrial function.

In line with these results, psoriatic fibroblast treatment with the SIRT1 inhibitor did not cause any significant change compared to psoriatic untreated cells. Moreover, we evaluated the mitochondrial superoxide production by confocal microscope analysis with the mitochondrial superoxide-specific fluorescent probe MitoSOX. MitoSOX is a selective indicator of mitochondrial superoxide, is selectively targeted to mitochondria and is able to efficiently compete with superoxide dismutase (SOD) for superoxide. Following mitochondrial oxidation, oxidized MitoSOX quickly migrates to the nucleus and becomes highly fluorescent upon binding to DNA. High levels of mitochondrial superoxide production in psoriatic fibroblasts compared to control cells are evident whereas SRT1720 treatment efficiently reverts this effect (Figure 4). In the presence of the SIRT1 inhibitor, high fluorescence, indicating high levels of mitochondrial superoxide, is evident. To further confirm and quantify these results, mitochondrial permeability transition pore opening, mitochondrial membrane polarization and mithocondrial superoxide production were also analyzed by flow cytometry (Figure 4B).

Mitochondrial number can influence experiments where mitochondrial function is investigated. We therefore counted mitochondria number in the SRT1720-treated or untreated fibroblasts from psoriatic patients (by confocal microscopy and FACS analysis) to ensure that altered mitochondrial function was not due to a mere numeric change of mitochondria. In our psoriatic fibroblasts, no significant variation in mitochondrial number upon treatment was evident (data not shown). Therefore, all of the observed mitochondrial function and cell viability alterations are not ascribable to changes in mitochondrial number.

### 2.5. SIRT1 Activation Protects Psoriatic Fibroblasts from Apoptosis

**Figure 5:**
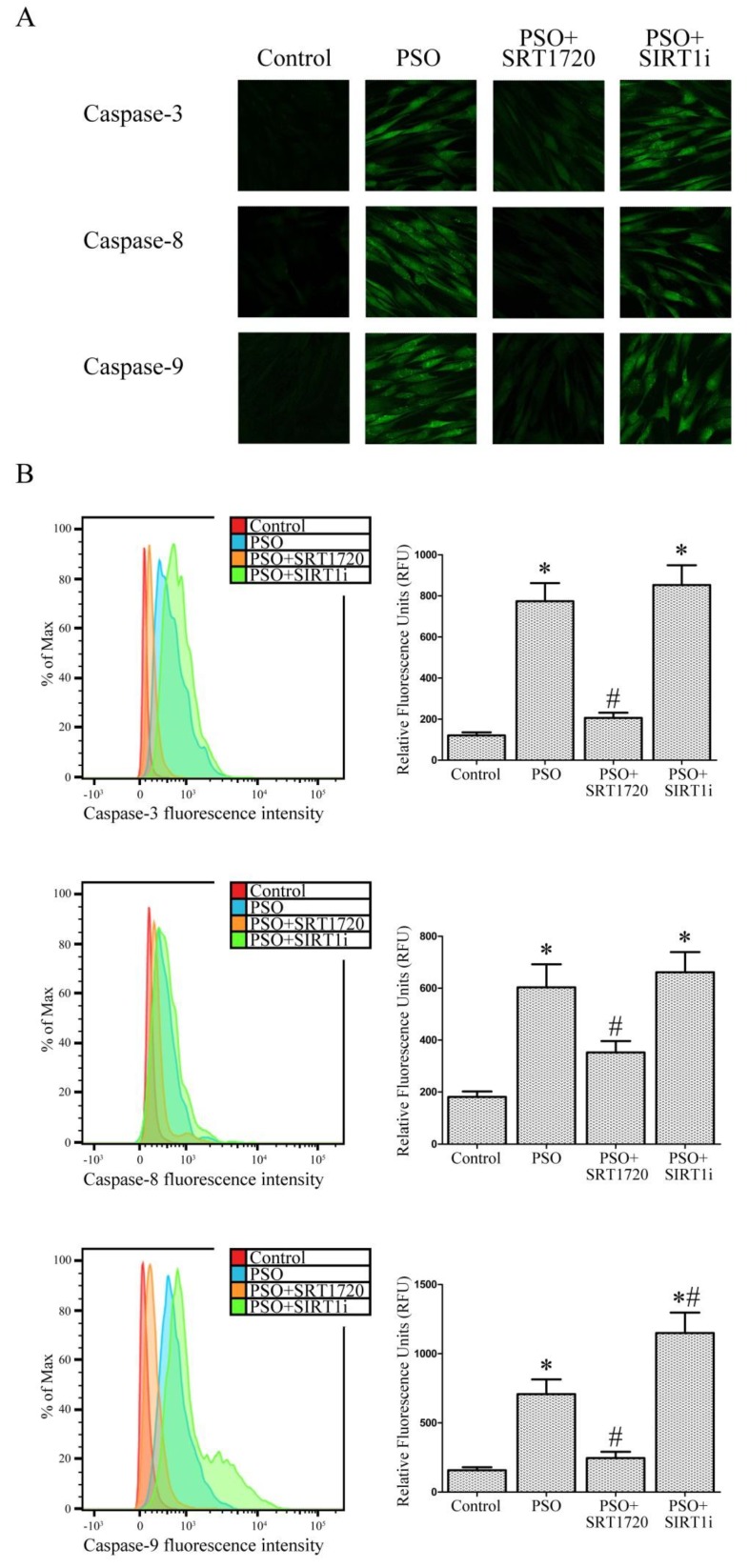
(**A**) Confocal microscope analysis (63× magnification) and (**B**) flow cytometry analysis of caspases-3, 8 and 9 activation in fibroblasts from controls (*n* = 4 biopsies) and psoriatic patients (*n* = 4 biopsies) in the presence of SRT1720 or the SIRT1 inhibitor (SIRT1i). Each experiment was performed in triplicate. * Significant difference (*p* ≤ 0.05) vs. fibroblasts from healthy patients. # Significant difference (*p* ≤ 0.05) vs. fibroblasts from psoriatic patients.

Caspase-3, -8 and -9 activities, which play a central role in apoptosis, were measured to verify the occurrence of apoptosis under our experimental conditions. As shown in Figure 5A, confocal microscope analysis clearly demonstrated marked caspase-3, -8 and -9 activities in fibroblasts from psoriatic patients and this increase was abrogated by SRT1720 treatment.

These important findings were also quantified by FACS analysis (Figure 5B). In particular, caspase-3 activity (Figure 5B) was strongly increased in psoriatic fibroblasts (+540% vs. fibroblasts from healthy subjects, *p* < 0.01) whereas SIRT1 activation strongly inhibited this effect (−376% vs. untreated PSO fibroblasts, *p* < 0.01). This effect was completely neutralized when using the SIRT1 inhibitor. Caspase-8 activity (Figure 5B), which plays a pivotal role in the extrinsic apoptotic signaling pathway via death receptors, was significantly increased in psoriatic fibroblasts (+233% vs. control fibroblasts, *p* < 0.01). SIRT1 activation clearly abrogated this effect (−71% vs. untreated PSO cells, *p* < 0.01). Once again, SIRT1 inhibitor treatment reverted this effect. Finally, we evaluated caspase-9 activity, a key player in the intrinsic or mitochondrial apoptotic pathway. As expected, fibroblasts from psoriatic patients showed increased levels of caspase-9 activity (+348% vs. control fibroblasts, *p* < 0.01) and SRT1720 treatment strongly reduced this effect (−188% vs. untreated PSO fibroblasts). Moreover, when SIRT1 signaling was inhibited, caspase-9 activity was even more marked than that observed in untreated PSO cells (+62% vs. untreated PSO fibroblasts, *p* < 0.01). All these findings point to a protective role of SIRT1 activation against apoptotic cell death in fibroblasts from psoriatic patients.

### 2.6. SIRT1 siRNA-Treatment of Fibroblasts from Healthy Subjects

**Figure 6:**
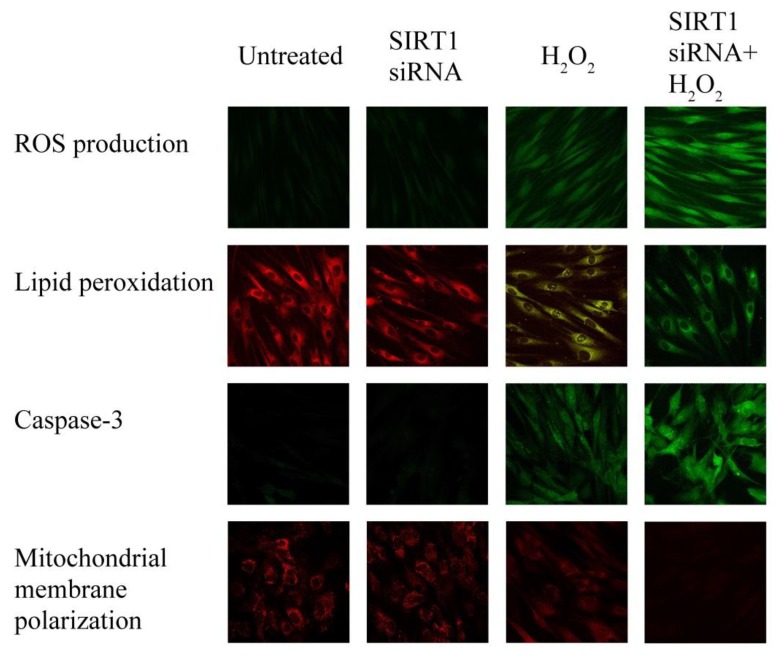
SIRT1 effects in oxidative stress protection. ROS production, lipid peroxidation, caspase-3 activity and mitochondrial membrane polarization were measured by confocal microscopy (63× magnification) in untreated and in SIRT1 siRNA-treated control fibroblasts, challenged or not with H_2_O_2_ (500 µM for 3 h) to elucidate the role of SIRT1 in oxidative-mediated cell injury. Fibroblasts were obtained from healthy skin (*n* = 4 biopsies). These parameters were measured in untreated and in SIRT1 siRNA-treated control fibroblasts, challenged or not with H_2_O_2_ (500 µM for 3 h).

ROS production, lipid peroxidation, caspase-3 activity and mitochondrial membrane polarization were measured in untreated and in SIRT1 siRNA-treated control fibroblasts, challenged or not with H_2_O_2_ (500 µM for 3 h) to elucidate the role of SIRT1 in oxidative-mediated cell injury. Without H_2_O_2_, the biochemical modifications displayed by SIRT1 siRNA-treated fibroblasts did not differ from untreated fibroblasts (Figure 6) in agreement with our previous data [21,22].

In the presence of H_2_O_2_ treatment, the observed alterations of the SIRT1 siRNA-treated fibroblasts paralleled those observed in fibroblasts from psoriatic skin (Figure 6). These findings suggest that psoriatic fibroblast damage can be mediated by SIRT1.

### 2.7. SIRT1 Activation Modulates MAPK Pathways in Fibroblasts from Psoriatic Patients

In our previous studies, we demonstrated the central role of MAPK pathways in inducing cell damage and apoptosis [21,22,24]. Here, we examined p38, ERK and JNK activation in response to SIRT1 activation.

**Figure 7:**
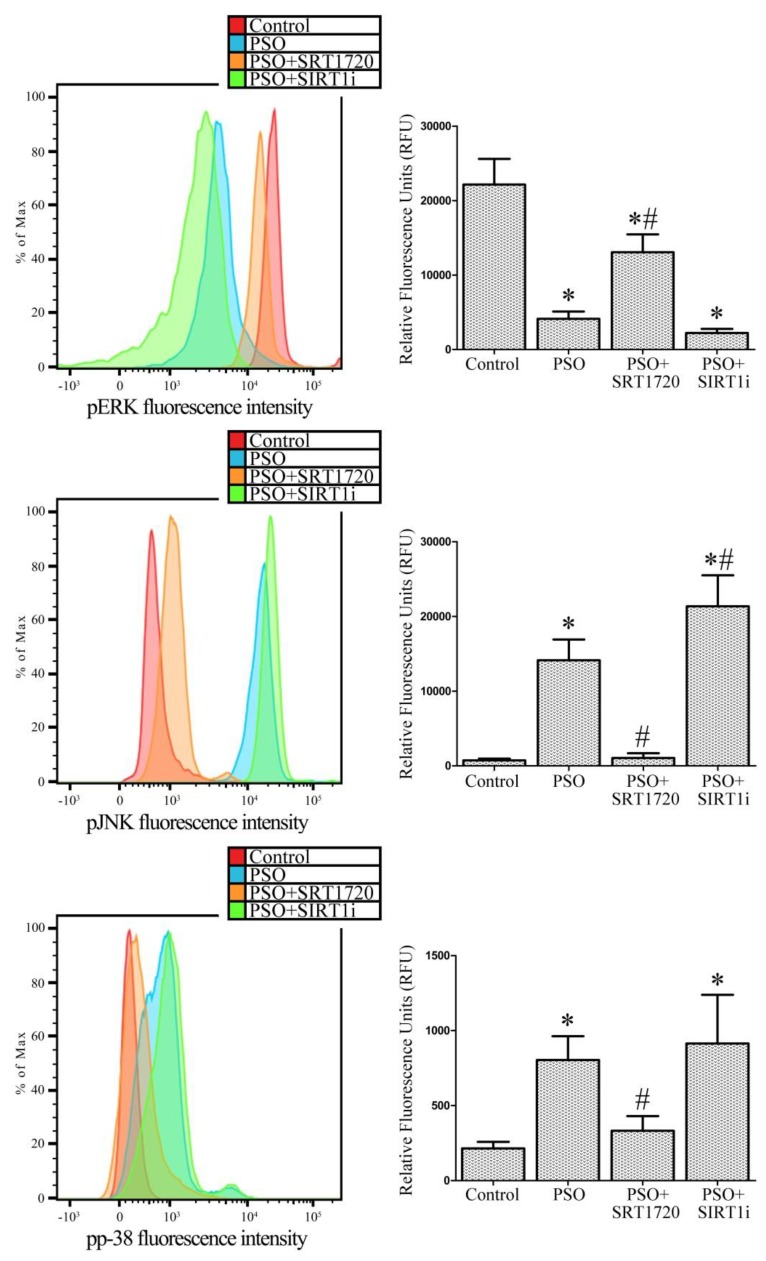
MAPK phosphorylation in fibroblasts from controls (*n* = 4 biopsies) and psoriatic patients (*n* = 4 biopsies) in the presence of SRT1720 or the SIRT1 inhibitor (SIRT1i). Each experiment was performed in triplicate. * Significant difference (*p* ≤ 0.05) vs. fibroblasts from healthy patients. # Significant difference (*p* ≤ 0.05) vs. fibroblasts from psoriatic patients.

In Figure 7, the levels of ERK phosphorylation, whose anti-apoptotic effect is well documented, are shown. In psoriatic fibroblasts, low levels of ERK phosphorylation were observed with respect to control fibroblasts (−437% vs. control, *p* < 0.01). SIRT1 activation induced a strong and significant increase in ERK phosphorylation (+217% vs. untreated PSO fibroblasts, *p* < 0.01) whereas SIRT1 inhibitor treatment significantly decreased ERK phosphorylation (−54% vs. untreated PSO fibroblasts, *p* < 0.01). At the same time, high levels of JNK phosphorylation were observed in psoriatic cells (about twenty-fold increase vs. the control, *p* < 0.01). SIRT1 activation completely abrogated JNK phosphorylation (−1340% vs. untreated PSO cells, *p* < 0.01) whereas the SIRT1 inhibitor led to an increase in JNK phosphorylation (+51% vs. untreated PSO cells, *p* < 0.01), demonstrating a role of SIRT1 in the JNK pathway.

p38 phosphorylation was also markedly enhanced in psoriatic fibroblasts (3.7-fold increase vs. control fibroblasts, *p* < 0.01). SIRT1 activation significantly reduced p38 phosphorylation (−142% vs. untreated PSO fibroblasts, *p* < 0.01) whilst the SIRT1 inhibitor completely abolished this effect (no significant difference vs. untreated PSO cells was evident).

**Figure 8:**
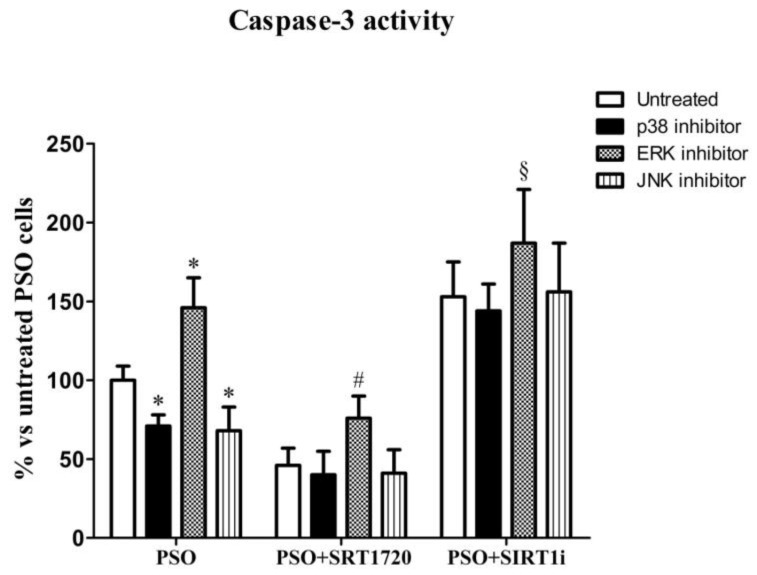
The caspase-3 activity measured using flow cytometry analysis in fibroblasts from psoriatic patients (*n* = 3 biopsies) in the presence of specific MAPK inhibitors. Each experiment was performed in triplicate. * Significant difference (*p* ≤ 0.05) vs. untreated fibroblasts from psoriatic patients. # Significant difference (*p* ≤ 0.05) vs. MAPK inhibitors untreated PSO + SRT1720 fibroblasts. § Significant difference (*p* ≤ 0.05) vs. MAPK inhibitors untreated PSO + SIRT1i fibroblasts.

To further investigate the role of SIRT1 in the molecular pathways of psoriatic fibroblasts, we measured caspase-3 activity in the presence of specific MAPK inhibitors. In Figure 8, we show that in the presence of p38 or the JNK inhibitor, caspase-3 activity significantly decreased (*p* < 0.01 vs. untreated PSO fibroblasts), indicating the involvement of these pathways in the induction of apoptosis in psoriatic fibroblasts. Remarkably, in the presence of ERK inhibitor, untreated fibroblasts displayed the highest caspase-3 activity, demonstrating the key role of ERK in the protection against apoptotic cell death.

Upon SIRT1 activation with the above inhibitors, a further decrease in caspase-3 activity was observed (*p* < 0.01 vs. untreated psoriatic fibroblasts). On the contrary, the SIRT1 inhibitor together with p38 or JNK inhibitors induced an increase in caspase-3 activity. Simultaneous treatment with the three inhibitors did not protect against cell death (data not shown). All these data demonstrated the key role of the SIRT1 pathway against apoptotic cell death via MAPK modulation.

## 3. Discussion

The present study was undertaken to explore the potential contribution of SIRT-1 signaling to the pathogenetic mechanisms of psoriasis. Both innate and adaptive immune systems play a central role in the disease which can be defined as an inflammatory skin disorder characterized by abnormal keratinocyte proliferation and differentiation [1,25]. The key role of dermal fibroblasts in regulating epidermal microenviroment, immune cell behaviour [26,27] and keratinocyte function has been already suggested [1,5,26,27]. Indeed, impaired fibroblasts can extensively produce superoxide and H_2_O_2_ (modifying the redox balance of psoriatic derma), promote inflammatory mechanisms [28] and may contribute to the epidermal overgrowth by inducing keratinocyte proliferation [29,30,31,32]. Based on this background, fibroblast cultures derived from psoriatic lesions represent our selected experimental model.

Mammalian skin is equipped with efficient antioxidant defence mechanisms that prevent oxidative injury to lipids and proteins, contributing to barrier integrity, which is essential for healthy skin condition. Thus, cellular redox balance is essential for skin homeostasis and an imbalance between pro-oxidant and antioxidant mechanisms can result in skin diseases, including psoriasis [33].

In the blood of psoriatic patients, redox imbalance has been witnessed by the presence of lipid peroxidation (increased levels of malondialdehyde) associated with decreased erythrocyte-superoxide dismutase activity, catalase activity and total antioxidant status [12,34]. In line with these observations, we recently identified NADPH oxidase as one of the possible upregulated ROS sources in lesional fibroblasts from psoriatic patients [35]. However, the molecular mechanisms underlying this alteration in psoriatic fibroblasts are still poorly understood.

Oxidative stress conditions and other metabolic and genotoxic stress are sensed and counteracted by sirtuins, a family of NAD^+^-dependent deacetylases which contribute to react to these damaging conditions through diverse pathways. Sirtuin 1 (SIRT1), in particular, is involved in the regulation of cellular survival, cellular senescence/aging, inflammation-immune function, endothelial functions, and circadian rhythms [21,22,36,37]. Moreover, SIRT1 is able to promote the differentiation of human keratinocytes [38] and its activation has been shown to inhibit keratinocyte proliferation [39]. Recently, some authors reported significantly decreased SIRT1 levels in lesional skin from psoriatic samples compared to controls, suggesting its potential involvement in the pathogenesis of psoriasis [40]. Furthermore, in a recent randomized, double-blind, placebo-controlled Phase IIa study, the effects of a selective small molecule SIRT1 activator in 40 patients with moderate-to-severe psoriasis were investigated [41]. On the basis of these observations and of our recent findings [23], here we demonstrate that, in fibroblasts from lesional psoriatic skin, SIRT1 activity is strongly reduced compared to healthy fibroblasts, suggesting a possible involvement of SIRT1 in the pathogenesis of psoriasis.

Another important finding, consistent with our previously obtained results [42], is the presence of oxidative stress in psoriatic fibroblasts, as suggested by enhanced lipoperoxidation and TAC reduction. Interestingly, psoriatic fibroblasts treated with the SIRT1 activator showed reduced oxidative stress levels, an effect that was reversed by the SIRT1 inhibitor, thus demonstrating a prominent role of SIRT1 in the maintenance of redox homeostasis. These findings are in line with a study reporting SIRT1-induced resistance to oxidative stress through FoxO in fibroblasts [19], where, upon SIRT1 overexpression, catalase expression was stimulated. In the presence of FoxO1a dominant negative, SIRT1 upregulation was inhibited [19]. The key role of SIRT1 in the protection against oxidative stress-induced cellular damage has been also confirmed by our experiments performed in SIRT1 siRNA-treated healthy skin fibroblasts.

In psoriatic fibroblasts, mitochondrial dysfunction, enhanced ROS production and signs of oxidative stress were present: indeed, mPTP opening and aberrant mitochondrial depolarisation were associated with significantly higher mitochondrial superoxide generation. Several studies indicate that SIRT1 activation reduces oxidative stress and maintains mitochondrial function. It has been reported that renal injury after ischemia-reperfusion is reduced by SIRT1, which is able to decrease nitrosative stress and inflammation and enhance energy metabolism by stimulating mitochondrial biogenesis [43]. An animal experimental model of hemorrhagic shock and reperfusion clearly demonstrated that SIRT1 activation induces p53 deacetylation, inhibits mPTP opening, suppresses a mitochondria-mediated apoptotic pathway and attenuates renal injury [44]. Other studies have also demonstrated that SIRT1 can stimulate the expression of antioxidants and, via an auto-feedback loop, can also potentiate SIRT1 expression [45,46,47,48,49]. Finally, it has been shown that SIRT1 overexpression protects human skin fibroblasts from UVB-induced cellular damage, increasing the resistance to oxidative stress [50]. Our results clearly demonstrate a protective effect of activated SIRT1 in the mitochondria of psoriatic fibroblasts where superoxide production, mitochondrial depolarization and impaired mPTP opening were inhibited by SRT1720 treatment. These findings are in line with our previous data demonstrating a protective role of SIRT1 in perilesional vitiligo keratinocytes [22], where oxidative stress and mitochondrial dysfunction were removed by treatment with resveratrol (a SIRT1 activator compound).

Our data clearly show that in psoriatic fibroblasts, the mitochondrial alterations (associated with an increase in ROS production) are responsible for the strong activation of the apoptotic process. In particular, caspase-3, -8 and -9, which play a key role in apoptotic cell death, displayed increased activities in psoriatic fibroblasts. Treatment with SRT1720 induced a significant downregulation of the apoptotic pathway and the presence of SIRT1 inhibitor counteracted this reduction suggesting a pivotal role for SIRT1 in the modulation of apoptosis in psoriatic fibroblasts.

To examine in depth the role of SIRT1 signaling in psoriasis, we performed experiments aimed at highlighting the relationship between SIRT1 and the redox-sensitive MAPK pathway, which has been shown to be altered in psoriasis [13,14,51,52]. MAPKs (mitogen-activated protein kinases) are signaling proteins activated by substantially diverse extracellular stimuli (cytokines, hormones, growth factors, UV irradiation, oxidative stress) and which regulate fundamental cellular processes (gene expression, cell proliferation, growth, survival, migration, and apoptosis) [53,54]. MAPKs are activated by phosphorylation of both threonine and tyrosine residues, and in turn they phosphorylate other downstream intracellular kinases and transcription factors. Extracellular signal-regulated kinase 1/2 (ERK1/2), p38 MAPK, and c-Jun N-terminal kinase (JNK) are the members of MAPKs whose involvement in oxidative stress signalling has been extensively investigated. JNK, ERK and p38 kinases exhibit distinct effects on apoptotic cell death. In the present study, using inhibitors for each kinase, we observed that JNK and p38 kinases promote apoptosis, while ERK displays anti-apoptotic effects. In particular, in psoriatic fibroblasts, we observed a decrease in p-ERK, which was reversed by SRT1720 treatment. In contrast, previous studies reported an increased ERK activation in human psoriatic lesion as compared with that in normal human epidermis [14] and in cell extracts from lesional psoriatic skin [13]. To further investigate the molecular pathways underlying the protective effects induced by SIRT1 activation, caspase-3 activity was measured in psoriatic fibroblasts in the presence of the ERK inhibitor, demonstrating the key role of ERK activation in protection against apoptotic cell death. In particular, the effect of the ERK inhibitor was suppressed by SRT1720 treatment, indicating a pivotal role of SIRT1 in protection against apoptosis. Our data are in agreement with previous studies reporting that SIRT1 activation promotes ERK phosphorylation in human fibroblasts [55] and that the histone deacetylase inhibitor suppresses the Ras-MAP kinase signaling pathway [56], suggesting that SIRT1 may stimulate the ERK pathway.

It has been demonstrated that JNK is not active in healthy human epidermis, but its activity is increased in psoriasis [17]; moreover, experimental data highlighted that TNF-α and UV light exert their pro-inflammatory effects in part via JNK activation [57,58]. In an elegant study, Gazel and co-workers demonstrated that JNK inhibition in epidermal keratinocytes is sufficient to initiate their differentiation, suggesting that attenuating JNK activity could be a differentiation therapy-based approach for treating psoriasis [59]. Here, we show a significant increase in JNK activity in psoriatic fibroblasts and interestingly, we report that SIRT1 activation prevents JNK activation. Since the p38 inhibitor prevents fibroblast apoptosis, a pivotal role for JNK MAPK in this pathway can be proposed.

The role of p38 MAPK in the development of psoriasis is well documented [13,14,52]. In particular, it has been shown that p38 MAPK activity is increased in lesional compared to non-lesional psoriatic skin [13]; moreover, the antimicrobial peptide S100A8, known to be upregulated in lesional psoriatic skin and believed to play a role in the pathogenesis of psoriasis, was found to be regulated by a p38 MAPK-dependent mechanism in cultured human keratinocytes [60]. p38 MAPK has been found to be activated in keratinocytes treated with H_2_O_2_, tumor necrosis factor (TNF)-α and interleukin (IL)-1β, suggesting a key role of p38 MAPK in mediating keratinocyte responses to cellular stress [61,62]. Because lesional psoriatic skin is characterized by increased expression of inflammatory cytokines [63], it is possible that p38 MAPK has a role in the inflammatory aspect of psoriasis. Interestingly, we found that p38 MAPK activity was upregulated in psoriatic fibroblasts compared to healthy fibroblasts and, upon SIRT1 activation, p38 activation was dramatically reduced. Moreover, in the presence of a p38 inhibitor we observed a significant reduction in apoptosis, confirming the key role of p38 MAPK in this pathway. Our data are in agreement with a previous study of Soegaard-Madsen and co-workers [52], demonstrating that p38 inhibition may be a mechanism by which the anti-TNF-α agent Adalimumab mediates its anti-psoriatic effect.

This study is not without limitations. First, the study population is small and it requires verification in a larger cohort (twelve patients and seven controls were enrolled in this study but each experiment is related to fibroblasts derived from three or four patients/controls only). In addition, the psoriatic subjects included in the study were characterized by moderate psoriasis (PASI = 12.4 ± 0.5). Therefore, the results need confirmation using a similar study design involving patients affected by severe disease. Another important consideration must be taken into account: even if fibroblasts and endothelial cells contribute to the pathogenesis of psoriasis, further studies on SIRT1 signaling are also needed on keratinocytes, which display a crucial role in the development of psoriatic lesions.

The involvement of SIRT1 in the protective mechanisms related to stress responses via its interactions with different substrates has been already studied [64] but data on skin biology are almost unknown. In particular, this is the first study exploring SIRT1 signaling in psoriasis. The present data demonstrate that in fibroblasts from psoriatic patients, SIRT1 activation displays an active role in restoring both mitochondrial function and redox balance via modulation of MAPK signaling. Therefore, SIRT1 can be proposed as a novel molecular target for the treatment of psoriasis.

## 4. Materials and Methods

### 4.1. Patients

The Local Ethics Committee approved the present study. All experiments were performed on twelve patients (six males and six females) affected by moderate psoriasis (PASI = 12.4 ± 0.5) with a mean age of 41.4 ± 7.6 years and with a mean disease duration of 14.1 years (from 8 to 25 years). The demographic and clinical data for each patient are summarized in Table 1. Seven healthy controls (four males and three females), matched for age and body mass index, were also enrolled in the study. No subject was subjected to any systemic therapy before or during the study, or had a history of any disease, e.g., diabetes mellitus and atherosclerosis, which might affect blood redox status. All subjects provided signed informed consent. The study was carried out according to the Helsinki Declaration.

### 4.2. Fibroblast Isolation and Setting Up of Cell Cultures

Lesional skin punch biopsies from twelve patients affected by plaque psoriasis and from seven healthy controls were used to obtain primary fibroblasts cell cultures. Briefly, skin biopsies were incubated with dispase II (2 U/mL, Sigma-Aldrich, Milan, Italy) and the derma was digested with collagenase (3 mg/mL, Sigma-Aldrich) for 45 min at 37 °C. Then, cells were filtered through a 70 μm filter to remove debris. Cells were cultured in DMEM medium (Sigma-Aldrich) with 10% FBS (Sigma-Aldrich) and 1% penicillin/streptomycin (Sigma-Aldrich). Fibroblasts were characterized by a high Vimentin (Sigma-Aldrich) expression by FACS and confocal microscopy analyses. Cells up to the fourth passage were used for experiments.

### 4.3. Preparation of Cell Homogenates

After trypsinization, fibroblasts (1 × 10^6^) were resuspended in 100 μL of lysis buffer (20 mM Tris-HCl pH8, 1% Triton X-100, 10% (*v*/*v*) glycerol, 137 mM NaCl, 2 mM EDTA and 6 M urea supplemented with 0.2 mM PMSF, 10 mg/mL leupeptin + aprotinin). Samples were then twice sonicated in ice for 5 s, centrifuged at 14,000× *g* for 10 min at 4 °C, and the supernatant collected. Protein concentration was determined according to the Bradford method [65].

### 4.4. Western Blot Analysis of SIRT1

SIRT1 expression levels were assessed by Western blot analysis. Briefly, equal amounts of homogenates (50 μg) were separated on 4–12% SDS-PAGE gels (Criterion XT, Bio-Rad Laboratories, Milan, Italy). After blotting into PVDF Hybond membranes and incubation overnight at 4 °C with (rabbit) anti-SIRT1 antibody (Santa Cruz Biotechnology Inc., Santa Cruz, CA, USA), membranes were washed and incubated for 1 h with peroxidase-conjugated secondary antibody. Then, bands detected with a SuperSignal West Dura (Pierce, Rockford, IL, USA) were quantified using Quantity-One software (Bio-Rad, Milan, Italy) [66]. SIRT1 expression levels were calculated as ratios between the densitometry of the corresponding band and the loading control (GAPDH).

### 4.5. Determination of Cellular SIRT1 Activity

SIRT1 activity was determined according to the method described by Fulco et al. [67] with some modifications. SIRT1 activity was determined by the SIRT1 Direct Fluorescent Screening Assay Kit (Cayman, Ann Arbor, MI, USA) as previously reported [22]. The fluorescence was detected using a Perkin-Elmer (Milan, Italy), LS 55 luminescence spectrometer (ex: 360 nm; em: 460 nm).

### 4.6. Cell Treatments

Fibroblasts from psoriatic patients or controls were grown for 24 h in the presence of 10 μM SRT1720 (a selective small molecule activator of SIRT1).

In another set of experiments, cells were treated with 10 μM p38 kinase inhibitor (SB203580), 10 μM JNK inhibitor (SP600125), 10 μM MEK inhibitor (PD98059), and 1 μM specific SIRT1 inhibitor (6-Chloro-2,3,4,9-tetrahydro-1*H*-Carbazole-1-carboxamide) for 3 h. All reagents were purchased from Sigma at the highest purity available.

### 4.7. Determination of Total Antioxidant Capacity (TAC)

As previously reported, intracellular TAC was measured in cell lysates by a chemiluminescent assay using an Abel Antioxidant Test Kit (Knight Scientific Limited, Plymouth, UK) [68]. The protein content was measured by the Bradford method [65].

### 4.8. Determination of Lipid Peroxidation

8-Isoprostane levels (lipid peroxidation index) were measured in cell lysates using the 8-isoprostane EIA kit (Cayman Chemical Co., Ann Arbor, MI, USA), following the manufacturer’s instructions. Moreover, lipid peroxidation was investigated by confocal microscopy using BODIPY 581/591 C11 (Life Technologies, Carlsbad, CA, USA) [69]. The fluorescent probe BODIPY 581/591 C11 shifts its fluorescence from red to green in the presence of oxidizing agents. Fluorescence was detected using a confocal Leica TCS SP5 scanning microscope (Mannheim, Germany) using a Leica Plan Apo 63X oil immersion objective and then projected as a single composite image by superimposition. Lipid peroxidation was also quantified by FACS analysis. Cells were incubated in DMEM with BODIPY 581/591 C11 (2 μM) for 30 min at 37 °C [70] and analyzed using a FACSCanto flow cytometer (Becton-Dickinson, San Jose, CA, USA).

### 4.9. Assessment of Intracellular ROS, NO Production and Mitochondrial Superoxide

After seeding on glass cover slips, fibroblasts were loaded for 15 min at 37 °C with the following fluorescent probes: MitoSOX (mitochondrial superoxide-specific probe, 3 μM), DAR-1 (NO probe, 1 μM) and H_2_DCF-DA (ROS production probe, 1 μM) all purchased from Life Technologies, Carlsbad, CA, USA. Cells were fixed in 2.0% buffered paraformaldehyde for 10 min at RT, and the fluorescence was detected by a Leica TCS SP5 confocal scanning microscope (Mannheim, Germany). Mitochondrial superoxide, NO and ROS generation were also monitored using the same fluorescent probes (MitoSOX: 0.5 μM; H_2_DCF-DA: 1 μM; DAR-1: 1 μM) by FACSCanto flow cytometer (Becton-Dickinson) [70].

### 4.10. Mitochondrial Number

MitoTracker Deep Red 633 (Life Technologies, Carlsbad, CA, USA) was used to stain mitochondria by confocal microscopy as previously described [21]. Mitochondrial number was also monitored by flow cytometry. Cells were incubated with MitoTracker Deep Red 633 (200 nM) for 20 min at 37 °C and immediately analysed by a FACSCanto flow cytometer (Becton-Dickinson).

### 4.11. Mitochondrial Permeability Transition Pore Opening

The fluorescent calcein-AM probe was used to assess mitochondrial permeability, an indicator of mitochondrial dysfunction and early apoptosis, as described by Petronilli et al. [24], albeit with minor modifications [71]. Calcein-AM, after entering the cell and following deesterification, emits fluorescence. Cell cobalt chloride co-loading quenches cell fluorescence except for mitochondria where cobalt cannot enter (living cells). In contrast, in the case of mitochondrial permeability transition pore opening (mPTP), cobalt enters mitochondria and quenches calcein fluorescence (apoptotic cells). Thus, decreased mitochondrial calcein fluorescence represents a measure of the extent of mPTP induction. Mitochondrial permeability transition pore opening was monitored by confocal microscopy and FACS analysis [71].

### 4.12. Mitochondrial Membrane Potential

Tetramethylrhodamine methyl ester perchlorate (TMRM) was used to assess the mitochondrial membrane potential. For confocal microscope analysis, cells were cultured on glass cover slips and loaded for 20 min at 37 °C with 100 nM TMRM (Life Technologies, Carlsbad, CA, USA). After fixing, cells were analyzed using a confocal Leica TCS SP5 scanning microscope (Mannheim, Germany). Mitochondrial membrane potential was also quantified by flow cytometry. Cells were incubated for 20 min at 37 °C with TMRM (100 nM) in DMEM and analyzed using a FACSCanto flow cytometer (Becton-Dickinson).

### 4.13. Determination of Caspase Activity

Caspase-3 and caspase-9 activity were analysed by confocal microscopy and FACS analysis. Fibroblasts were loaded with FAM-FLICA™ Caspases solution (Caspase FLICA kit FAM-DEVD-FMK) for 1 h at 37 °C, washed twice with PBS and analysed by a Leica TCS SP5 confocal laser scanning microscope and FACSCanto flow cytometer (Becton-Dickinson) [72]. In another set of experiments, aimed to assess the role of ERK, p38 and JNK signalling pathways, cells were treated with 10 μM SP600125 (specific JNK inhibitor), 10 μM PD98059 (MEK inhibitor) or 1 μM SIRT1 inhibitor for 3 h prior (or not) to SRT1720 treatment.

### 4.14. SIRT1 RNA Interference (RNAi) Experiments

Fibroblasts from healthy subjects were cultured in complete medium without antibiotics for 2 days. Cells were seeded into a six-well plate. Then, 8 μl of LipofectamineTM LTX and 3 μL PLUSTM Reagent (Life Technologies, Carlsbad, CA, USA) were diluted in 90 μL of culture medium. Subsequently, 12 μL SIRT1 siRNA (siRNA for SIRT1-sc-40986- from Santa Cruz Biotechnology) was mixed with the medium containing Lipofectamine together with PLUS reagent and incubated for 30 min at RT for complex formation. Finally, cells were incubated with a final SIRT1 siRNA concentration of 100 nM. After 48 h, SIRT1 protein expression was determined by Western blot. To study the possible role of SIRT1 in the oxidative-mediated cell injury, untreated and SIRT1 RNAi-treated fibroblasts obtained from healthy subjects were challenged for 3 h with 500 μM H_2_O_2_. ROS production, lipid peroxidation, caspase-3 activity and mitochondrial membrane polarization were evaluated by confocal microscope analysis.

### 4.15. Assessment of MAPK Activity by FACS Analysis

Fibroblasts were fixed and permeabilized using the BD Cytofix/Cytoperm buffer (Becton-Dickinson) following the manufacturer’s instructions. Anti-Phospho-p38 MAPK (Thr180/Tyr182) (28B10) Mouse mAb (Alexa Fluor^®^ 488 Conjugate), anti-Phospho-SAPK/JNK (Thr183/Tyr185) (G9) Mouse mAb (PE Conjugate), and anti-Phospho-p44/42 MAPK (Erk1/2) (T hr202/Tyr204) (D13.14.4E) XP^®^ Rabbit mAb (Alexa Fluor^®^ 488 Conjugate) were used at 1:50 dilution for 1 h at RT according to manufacturer’s instructions.

### 4.16. Statistical Analysis

All data are expressed as the mean ± SD. Comparisons between groups were analyzed using one-Way Analysis of variance (ANOVA) followed by the Bonferroni *t*-test. A *p* value <0.05 was accepted as statistically significant.

## Figures and Tables

**Table 1:** Demographic and clinical data of psoriatic patients involved in the study.

Patient	Age	Body Mass Index (BMI)	Duration of Disease (Years)	PASI
M1	33	25	8	13
M2	41	24	12	13
M3	29	25	10	12
M4	52	23	21	12
M5	48	26	11	12
M6	45	25	10	13
F1	51	21	25	13
F2	45	23	20	13
F3	38	26	18	12
F4	32	24	12	12
F5	37	25	9	12
F6	46	26	13	12
Mean ± SD	41.4 ± 7.6	24.4 ± 1.5	14.1 ± 5.5	12.4 ± 0.5
